# Characteristics of pulsatile flows in curved stenosed channels

**DOI:** 10.1371/journal.pone.0186300

**Published:** 2017-10-19

**Authors:** Hyeonji Hong, Eunseop Yeom, Ho Seong Ji, Hyun Dong Kim, Kyung Chun Kim

**Affiliations:** 1 School of Mechanical Engineering, Pusan National University, Busan, South Korea; 2 MEMS Technology Centre, Pusan National University, Busan, South Korea; Coastal Carolina University, UNITED STATES

## Abstract

Spatial and temporal variations of the hemodynamic features occur under pulsatile conditions in complex vessel geometry. Wall shear stress affected by the disturbed flow can result in endothelial cell dysfunction, which leads to atherogenesis and thrombosis. Therefore, detailed understanding of the hemodynamic characteristics in a curved stenosed channel is highly important when examining the pathological effects of hemodynamic phenomena on the progression of atherosclerosis. The present study measures the velocity fields of pulsatile flows with three different Reynolds numbers in 3D curved vessel models with stenosis using time-resolved particle image velocimetry (PIV). Three different models were cast in PDMS polymer using models made by a 3D printer with different bend angles of 0°, 10°, and 20° between the longitudinal axes at the upstream and downstream of the stenosis. To investigate the 3D flow structures, a stack of 2D velocity fields was obtained by adjusting the position of the laser sheet along the Z-direction. The structures of flow fields in the stenosed models were analyzed using the distribution of the shearing strain as well as the skewness and full width at half maximum of the velocity profile. To support experiment results, distributions of pressure and 3D vortex in the curved stenosed channels were estimated by conducting the numerical simulation. These results indicate that the curvature of the tube considerably influences the skewness of the flow, and the shear stress is intensified near the outer curvature wall due to centrifugal force. The results would be helpful in understanding the effects of geometrical factors on plaque rupture and severe cardiovascular diseases.

## Introduction

Cardiovascular diseases (CVDs) such as atherosclerosis are a leading cause of death [1]. Atherosclerosis is a complex disease characterized by thickening of the intima (plaque). As it progresses, the plaque accumulated inside the arteries contributes to block blood flows to downstream tissue and eventually leads to plaque rupture. This progression is influenced by complex interactions between biological and mechanical factors [2]. In many previous studies, it has been suggested that hemodynamic and hemorheological features are closely related to cause, progress, and prognosis of atherosclerosis because wall shear stress (WSS) intensity, exposure time, and turbidity have effects on the structure and function of the endothelium [3, 4]. Specifically, low and oscillating WSS, affected by the disturbed flow, may result in endothelial cell dysfunction, which leads to atherogenesis and thrombosis [5].

When vessel geometry is stenosed, branched and bent shape, complex spatial and temporal WSS distributions are observed [2, 6]. Particularly, stenotic geometry produces regions with high and low WSS, flow separation, and recirculation [7]. These flow disturbances are related to biophysical properties such as stenosis severity, eccentricity, and ulceration [8]. Specifically, low WSS observed at the downstream of plaque shoulders, results in the progression of atherosclerosis [9]. Whereas High WSS proximal to the stenotic throat contributes to thinning of the fibrous cap and plaque rupture [10]. Thus, investigation of the geometric structures and hemodynamic features is important to understand the roles of WSS on CVDs, including the development of secondary stenosis [11]. Numerical analyses have been reported for estimating the flow in a curved channel [12–16]. The curvature results in centrifugal force and induces considerable secondary flows, which affect the reverse flow at the inner wall and skewness towards the outer wall [12, 17, 18].

Blood flows in the arteries of living beings are unsteady pulsatile flows, which are different from steady flow [19–21]. The instability in a stenosed vessel is dependent on the unsteady acceleration or deceleration in pulsatile flows due to the Coanda effect [22]. WSS and time-dependent vortex structures for pulsatile flows were compared with those of steady flow [19, 20]. Depending on the acceleration phase (systole) and deceleration phase (diastole) in pulsatile flow, the vortex is formed and broken down around the post-stenosed region. A small reverse flow also occurs at the vessel wall [23, 24].

Flow patterns in the curved vessels can be illustrated in detail through the velocity profiles or distribution with respect to position or time. Pedersen et al. (1993) described the relationship between the pulsatile inlet flow and the skewness of 2-dimensional flow based on the experimental velocity distribution. Perktold et al. (1991) and Krams et al. (2005) investigated the effects of the curved structure of the vessel model on the flow field through the velocity distribution using numerical and experimental analyses, respectively. Additionally, Keshavarz-Motamed et al. (2011) mentioned that profiles of velocity, pressure, and WSS have a relationship with factors such as the inlet flow conditions and curvature.

Previous studies have investigated the fluid characteristics around the curvature under pulsatile flows [12, 13, 15, 17, 25], or in curved channel with stenosis in steady flows [14, 16]. However, the results under steady flow conditions are quite different from those under pulsatile conditions when the flow passes through the stenosed channels. Previous studies investigating pulsatile flows in curved stenosed model reported only using numerical simulation [15]. The objective of this study is to measure the pulsatile flow in a curved channel with stenosis, quantitatively to analyze the behavior and structures of the flow over time, and to determine correlations between blood flow and several factors, such as the flow rate conditions and vessel geometries (degree of curvature). For that, the velocity profile and shear rate distribution were analyzed to describe different flow structures. Using a scanning process, the 3D flow structures around the stenosed channels were compared with respect to the degree of curvature. In addition to the experiment, numerical simulation was carried out to estimate distributions of pressure and 3D vortex in the curved stenosed channels.

## Materials and methods

### Experimental setup

**Fig 1:**
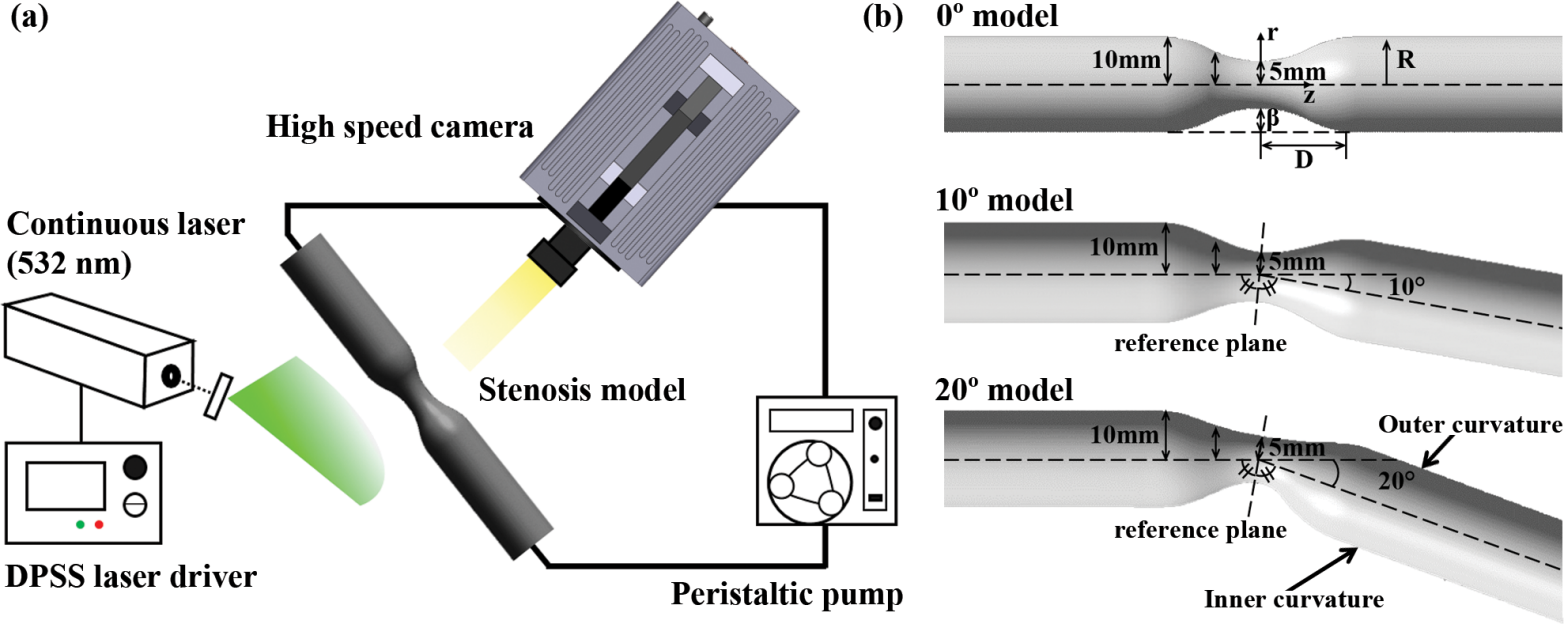
Schematics for the experiment. (a) Setup for PIV measurements. (b) three types of channels with stenosis using 3D modeling program. From top to bottom: 0° model (straight channel), 10° bent model and 20° bent model (curved channel).

The left side of **Fig 1** depicts a schematic of the experimental setup consisted of a PDMS stenosis model, peristaltic pump, continuous laser, and high-speed camera. The experiment was conducted at constant temperature (25°C). The peristaltic pump (WT600-2J; pump head: KZ25, tubing size: 36#) circulates the working fluid with different flow profiles from 1620 to 3660 mL/min, which depends on the RPM setting. Diode-pumped solid-state laser (DPSSL; wavelength: 532 nm) was used to generate a laser sheet with optics. Flow images in the stenosis model (1,024 × 1,024 pixels) were consecutively captured by using a FASTCAM SA1.1 (Photron, USA) at 5,400 frames per second (fps) to measure the velocity vector field.

### Fabrication of stenosed channel

Stenosed blood vessel models were made by a 3D printer. The printed core model was combined with an acrylic cavity mold, and polydimethylsiloxane (PDMS) was poured into the acrylic mold. Trapped air bubbles in the PDMS were removed, and the PDMS was cured using a vacuum oven (OV-11, JEIO TECH), in which the temperature and vacuum conditions were controlled. The printed core model and acrylic mold were removed from the hardened PDMS to complete the fabrication of the transparent PDMS block with the stenosed channels.

As shown in the right side of **Fig 1B**, three types of stenosed models have 50% severity (β) with different bend angles (0, 10, and 20°) between the longitudinal axes at the upstream and downstream of the stenosis [26, 27]. The stenosis in the middle of the channel has a sinusoidal shape by the following cosine-form equation.

$$\frac{r(z)}{R}=1-\frac{\beta }{2}\left[1+\cos\left(\frac{z\pi }{D}\right)\right],-D\leq z\leq D$$

where r and Z represent the radial and axial coordinates, and R and D indicate the radius and the diameter of the stenosed model. β is degree of occlusion, called severity in this section. The diameters of the channel and stenosis apex are 20 and 10 mm, respectively. When the modeling process was conducted, the shape of pre-stenosis part in three types of stenosed models was equally fixed for inducing the same flow behavior passing through ascending stenosis part. Then, the pre-stenosis part was mirrored with respect to each reference plane depending on the degree of curvature.

### Blood-mimicking working fluid

Silicone elastomer generally has a refractive index (RI) from 1.40 to 1.43. Mismatching of the RI between the channel and working fluid can lead to optical distortion. To match the RI between PDMS and working fluid, the working fluid was made by mixing glycerol and water, which have RIs of 1.47 and 1.33, respectively [28, 29]. Mass ratio of mixture between glycerol and water was 63:37, which was determined by observing the disappearance of optical distortion. The viscosity of the working fluid is about 16.0 cP [30].

### Particle image velocimetry (PIV)

In this study, time-resolved PIV was performed to measure the velocity variation over time due to the pulsatile characteristics. To investigate the 3D flow patterns, the 2D velocity field was measured at different heights by moving the laser light sheet upward from the center plane with the interval of 1.5 mm. PMMA-Rhodamine B particles (1–20 μm, 200 ml water suspension (50 g particles)) were used as tracer particles, which have a maximum excitation wavelength of 550 nm and emission wavelength of 590 nm. The size of each interrogation window was 64 × 8 pixels with 50% overlapping for all experimental conditions. Post-processing was done using in-house PIV programs to eliminate error vectors in the PIV results. The vector fields and contours of the shear rate were plotted using Tecplot 360 (Tecplot, Inc., Bellevue, WA), and a 3D plot was obtained using Matlab software (Mathworks, USA). All velocity data represent phase-averaged data averaged over 25 cycles.

### Numerical simulation

The numerical solutions of the fluid flow equations were carried out using CFX 16.1 (ANSYS, Inc., USA). In this analysis, the flow was regarded as laminar, and the vessel walls were assumed to be rigid. To obtain equation for transient mass flow rate, the experimental results for the case of average Re = 160 were fitted. Waveforms of experimental data and fitted mass flowrate are shown in the upper right side of **S1 Fig**. The obtained equation of mass flow rate was set at the inlet of the channel. Open conditions with a relative pressure of 0 Pa was employed at the outlet of the channel. No-slip condition was applied to every wall. To minimize the influence of initial flow conditions, all simulations were carried out for eight cycles. To demonstrate simulation data, simulation and experiment results were compared (**S2 Fig**). Velocity profiles at φ = 0.5 in upstream of stenosis obtained by experimental and simulation were well matched with high correlation coefficient (R^2^ = 0.985). For systematic analysis, flow rate measured by simulation and estimated by fitting experimental velocity profile were compared in Bland–Altman plot. The difference between two techniques is depicted against their average values. A bold line and dashed lines indicate the mean value and ± 95% limits of agreement, respectively. Considering that the presentation of data inside the 95% limits is utilized to judge the agreement of two techniques, the simulation results are in a good agreement with the experimental results.

## Results and discussion

### Pulsatile flow conditions

Due to the pulsatility of blood flow, the flow rate and pressure vary cyclically with respect to time. The continuously varying velocity at upstream of the stenosed channel can be transitional or turbulent [10]. To define blood flow in the present study, Reynolds number is calculated using the time-averaged velocity of blood flow (Re number = 160, 260, and 360). The results obtained at different Re numbers were compared to each other to analyze the effect of the inlet velocity conditions on the velocity, WSS, and flow patterns. The Re number increases as the volume flow rate is increased with increase in the rotational speed of the peristaltic pump. As shown in the **Table 1**, the corresponding Womersley numbers have the values within the range of 23–35 (α = 0.5D (ωρ/μ)1/2 where ω = 2π*f*). Gharib, M. and M. Beizaie (2003) reported that cardiac output increases when the heart rate is higher based on the data from the previous investigations and conducted experiment under α = 15.9–39 including exercise conditions [31]. In addition, for Womersley parameters above 10, the effects of α are not significant due to dominant unsteady inertial forces [32].

**Table 1:** The values related to the experimental conditions. From top to bottom, details about the stenosed model, working fluid and flow conditions.

	**Glycerol–Water Mixture (25°C)**		
**Density, ρ [kg/m**^**3**^**]**	**1150**		
**Viscosity, μ [Pa∙s]**	**1.11×10**^**−2**^		
	**Flow conditions**		
**Average Reynolds number Re**_**ave**_^**a**^ **(inlet)**	**160**	**260**	**360**
**Maximum Reynolds number Re**_**max**_^**b**^ **(flow)**	**1533**	**2072**	**2321**
**Womersley number, α**	**23**	**29**	**35**

^a^ Re_ave_ from the time-averaged inlet flow velocity

^b^ Re_max_ was calculated from the instantaneous velocity measured in the channel

**Fig 2:**
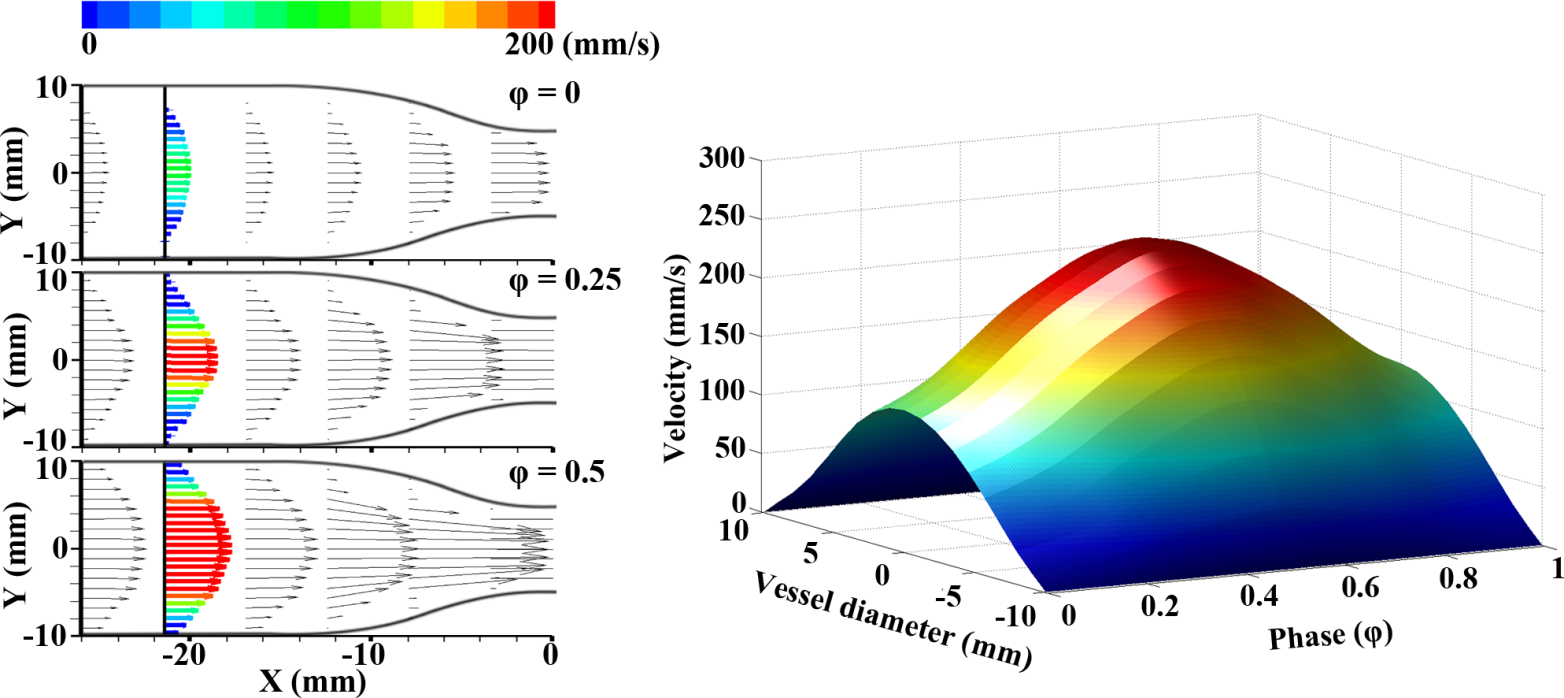
Pulsatile inlet flow velocity. The velocity vector fields (left panel) indicate the instantaneous velocity of fully-developed inlet flow at different phases of φ = 0, 0.25, and 0.5. Phase variation of velocity profile (right panel) obtained around -20X.

However, cyclic time for pulsatile flow is also varied from 8.1 to 18.3 Hz due to increase of rotational speed. The period of pulsatile flow becomes shorter at higher Re number. Since the flow profile periodically varies with respect to time, the time is normalized by the phase. The left-side of **Fig 2** shows the vector fields at upstream of the stenosis at representative phases of φ = 0, 0.25, and 0.5 for a Re number of 160. To describe the flow variation with respect to the phase, the radial velocity profile at a specific longitudinal position (X = -21.5 mm from stenosis apex) of the tube was extracted over time (right-side of **Fig 2**). A non-slip assumption was applied to obtain the velocity profile from the cross-sectional line. A phase of φ = 0 corresponds to the starting point of the acceleration. The inlet velocity increases for the acceleration section (systole) and reaches a peak value at φ = 0.5. And then, the deceleration section (diastole) is observed from φ = 0.5 to 1.

### Flow pattern in stenosed channels

**Fig 3:**
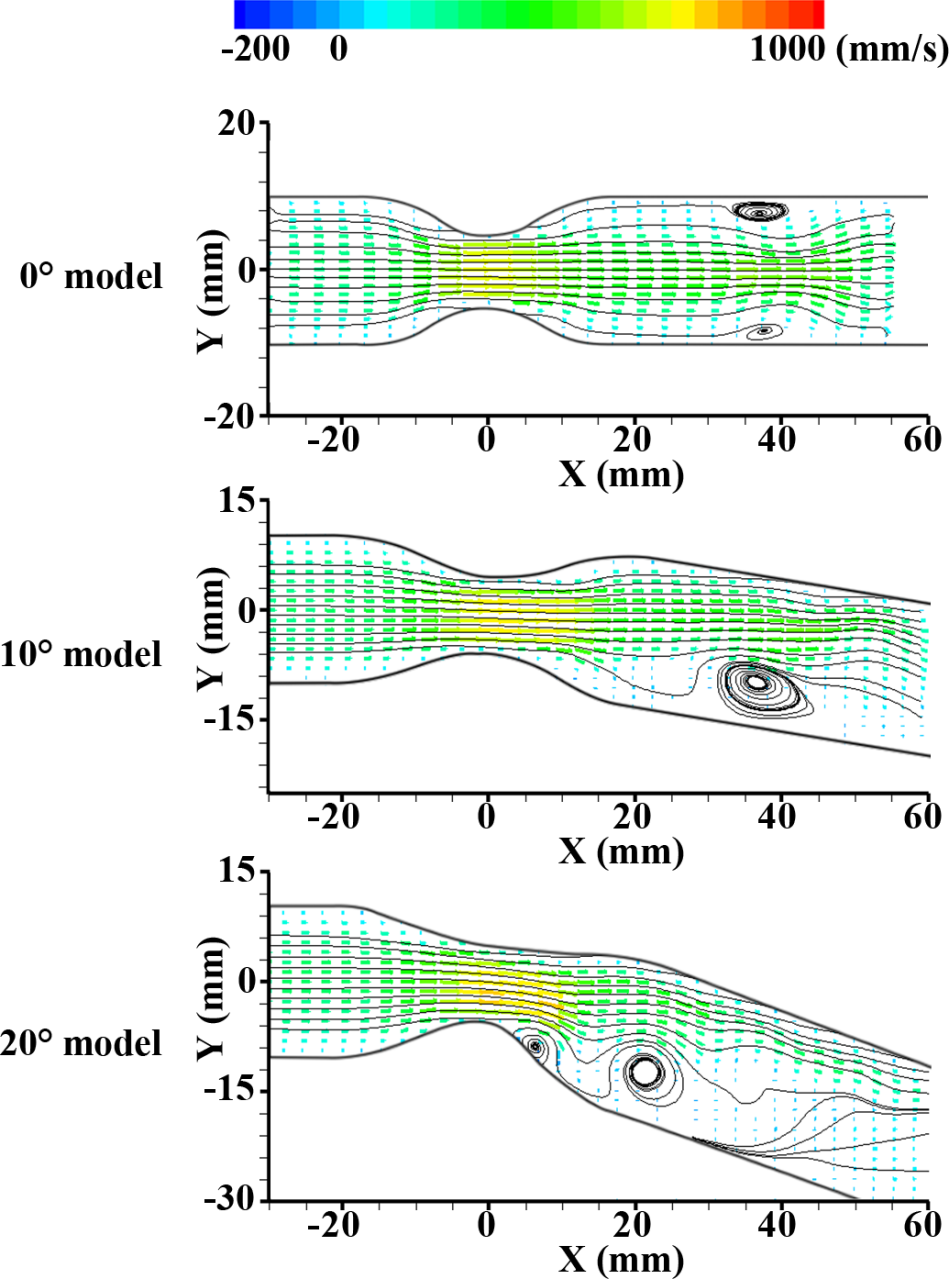
Instantaneous velocity vector fields at φ = 0.5 (peak inlet velocity) at Re = 160. From top to bottom: 0° model, 10° bent model, and 20° bent model.

**Fig 3** shows the velocity vector fields in different channel geometries at Re = 160. Each image is an ensemble-averaged field from about 100 images at the same phase (φ = 0.5; peak velocity in the phase). Recirculation occurs in the post-stenosis region due to pulsatile flow in all cases.

In the case of the 0° model, a symmetrical recirculation zone is generated at the downstream of the stenosis (X = 40 mm). The main stream flows parallel to the center of this channel. In the 10° model, a recirculation zone was also generated after the stenosis but mainly observed at the inner curvature with an asymmetrical distribution. Although the main flow at the upstream of the stenosis is parallel to the center axis of the channel, centrifugal force skews the flow towards the outer curvature of the downstream due to the bend angle of the curved channel. For that reason, the size of the recirculation zone for the 10° model is bigger than that for 0° model.

As expected, the noticeable centrifugal force for 20° model induces very skewed main stream toward the outer curvature and recirculation zone around the inner curvature. The accelerated velocity region is skewed towards the outer curvature wall and flows along the wall. This intensive centrifugal force due to curvature makes the wide flow separation. Then, the strong reverse pressure gradient is generated at the inner wall [15] and it tends to hold the vortex near stenosis [33]. Also, period of the pulsatile flows is short for recovering the pressure drop entirely after the stenosis. Therefore, in **Fig 3**, the generated vortex in 20° model stays around X = 20 mm at φ = 0.5, while vortices in other models appear around X = 40 mm. It means that the strong reverse flow at the inner curvature in the 20° model tends to hold the vortex near stenosis. As a result, the asymmetry of the flow patterns is pronounced with the bend angle increases. In other words, the forward jet flow is most skewed toward the outer curvature wall in the 20° model.

**Fig 4:**
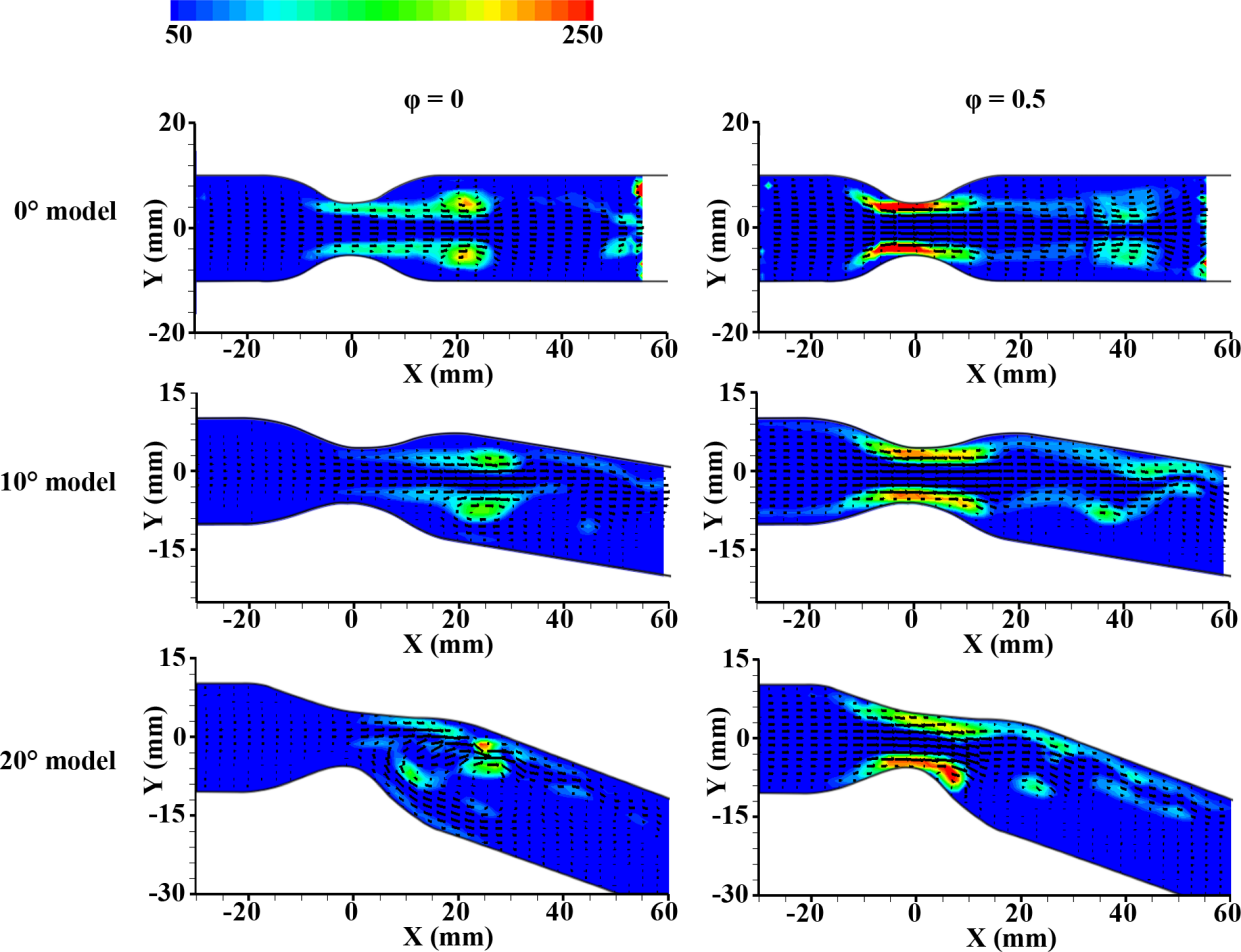
Ensemble-averaged velocity fields with the contour map of vorticity in the cases of 0°, 10° and 20° models (from top to bottom) at φ = 0 and 0.5. The inlet velocity is minimum at φ = 0 and is maximum at φ = 0.5.

**Fig 5:**
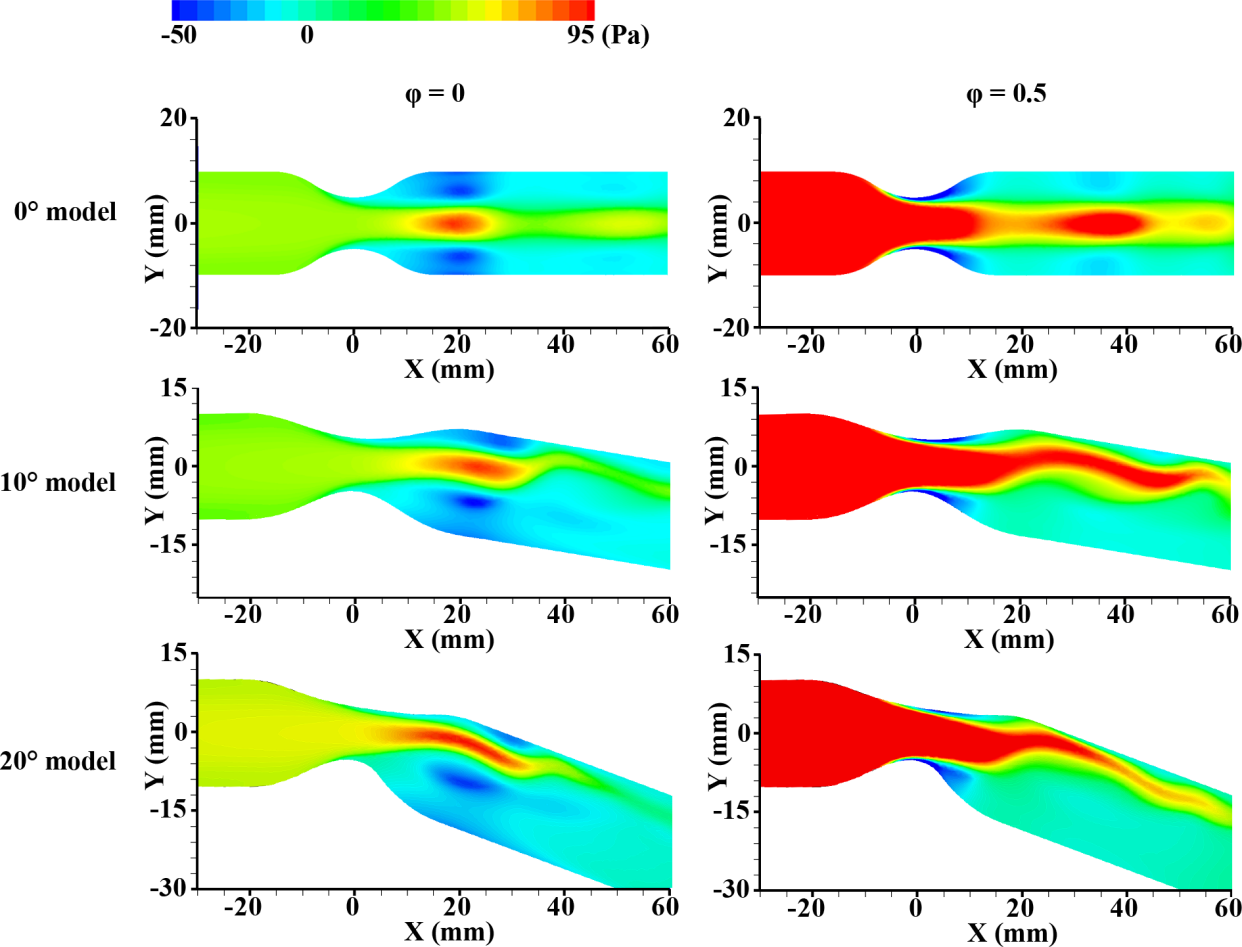
Simulation result depicting the distribution of total pressure in the cases of 0°, 10° and 20° models (from top to bottom) at φ = 0 and 0.5. The inlet velocity is minimum at φ = 0 and is maximum at φ = 0.5.

The vorticitis from experiment were compared with the pressure distributions from simulation data at the bottom phase velocity (φ = 0) and peak phase velocity (φ = 0.5) in **Figs 4 and 5**. At φ = 0, the negative pressure exists at the wall of post-stenosis as expected. The generated symmetric vortex has relatively high strength at this position in consequence of the reverse pressure gradient. However, at φ = 0.5, this vortex continuously has moved downstream and the magnitude of vortex weakens because the positive pressure was recovered from the negative due to flow acceleration. As shown in **Fig 3**, the accelerated velocity component is observed around the apex of stenosis and downstream vortex. The regions of high pressure in the simulation results correspond with the regions of the relatively high velocity region in the experiment results. After that, the vortex may be generated at the concavity (after the stenosis) again due to periodic pulsatile flow. These results infer that adverse pressure gradient caused by the stenosed apex induces flow separation at downstream of the stenosis, and the vortex generated at the concavity after the stenosis becomes larger in the diastole phase [34]. In contrast to 0° model, asymmetric vortex and pressure distribution with the relatively high backward flow at the inner curvature are observed in 10°, and 20° models. It is mainly caused by the shift of axial velocity towards the outside curvature. As the bend angle increases, the high centrifugal force leads to more skewed vortex and pressure distribution. In all phase, the asymmetric vortex generated after the stenosis at φ = 0 moves downstream periodically. The repetitive process was represented in **S1–S3 Movies 1–3**.

**Fig 6:**
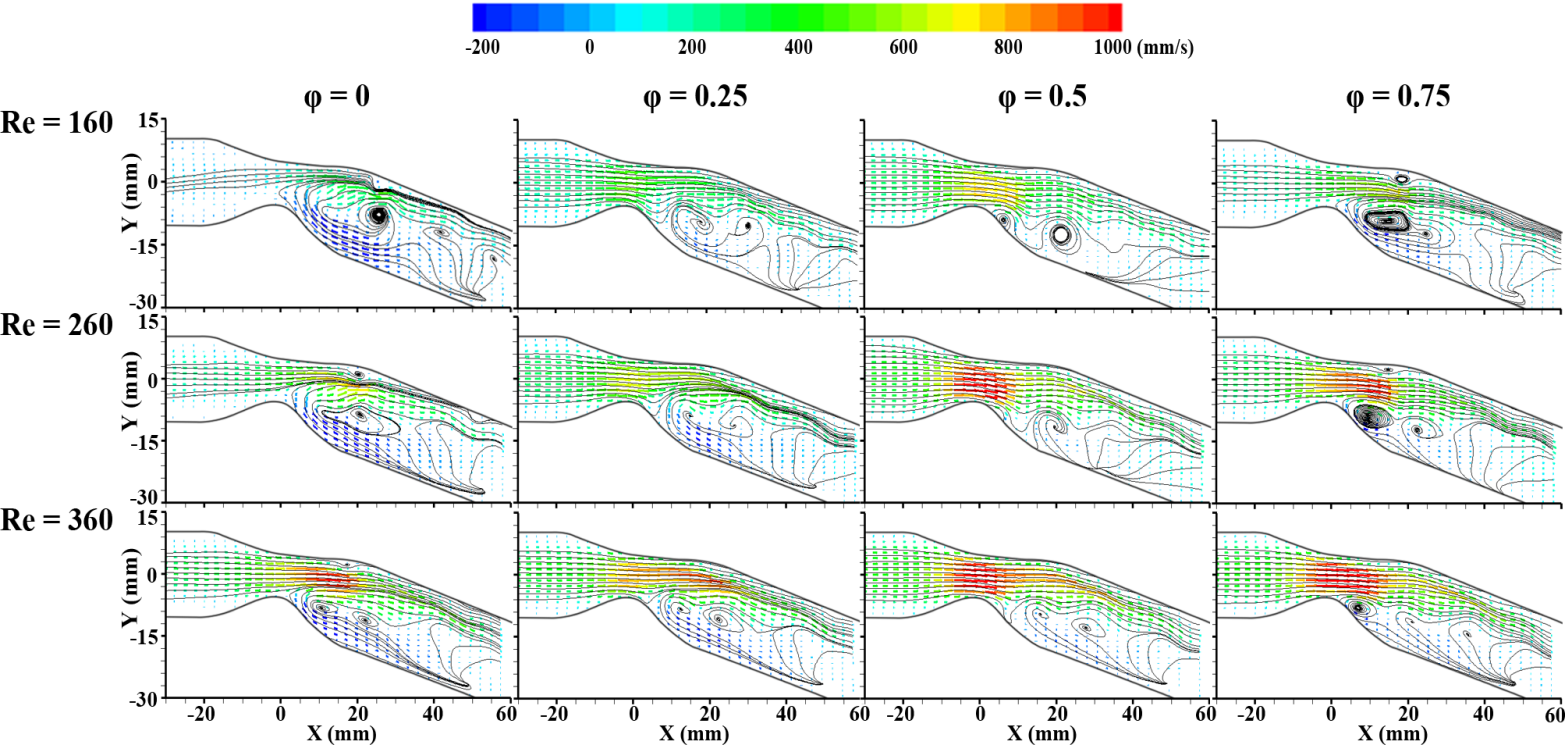
Instantaneous velocity vector fields in 20° bent model illustrating flow patterns such as the generation or movement of a vortex, and forward and backward flow regions, and different phases and Reynolds numbers. Reynolds numbers are 160 (top), 260 (middle) and 360 (bottom) with different phases of φ = 0, 0.25, 0.5, and 0.75.

**Fig 6** shows the characteristics of pulsatile flows at Re = 160, 260, and 360 in the 20° model. It is instantaneous vector fields for four representative phases of φ = 0, 0.25, 0.5, and 0.75. The period of the pulsatile flow decreases at higher Re number. Therefore, one specific pulsatile flow is accelerated by the subsequent pulsatile flow due to the short period at high Re number.

As the Re number increases, the acceleration region with high velocity generally increases around the stenosis apex. In the case of Re = 160, the main stream at the downstream of the stenosis is narrowest along the radial position, and a stronger reverse flow occurs at the inner curvature wall compared with the other two cases. The reverse flow is developed at the downstream during the deceleration phase [17]. In the case of Re = 360, the reverse flow at the inner curvature wall tends to keep its pattern overall in all four phases. This may result from the short period of the pulsatile flows because there is insufficient time to recover the pressure drop after the stenosis. Furthermore, the main streamline tends to retain its flow direction parallel to the tube wall. The interaction between axially oriented forces and centrifugal forces may have influence the kinetics of the fluid elements moving in the curved channel. The centrifugal force induces secondary velocity components, while the axially oriented forces induce main flow stream [15, 16].

During the early diastole phase (φ = 0.5–0.75), the maximum velocity region seems not to move as far downstream for all Re numbers. Acceleration occurs in the region when passing the stenosis. However, the curved geometry obstructs the accelerated flow path on the side of the outer curvature wall. It means that the forward flow region will change its direction less rapidly due to the curvature and inertia. In addition, the strong adverse pressure gradient can disturb the forward flow. From this reason, the maximum velocity region seems to stay within the range of X = 0 to 15 mm. Therefore, in the 20° channel, the movement of the maximum velocity region is short during deceleration despite the accelerated flow.

### Analysis of velocity profiles in stenosed channels

**Fig 7:**
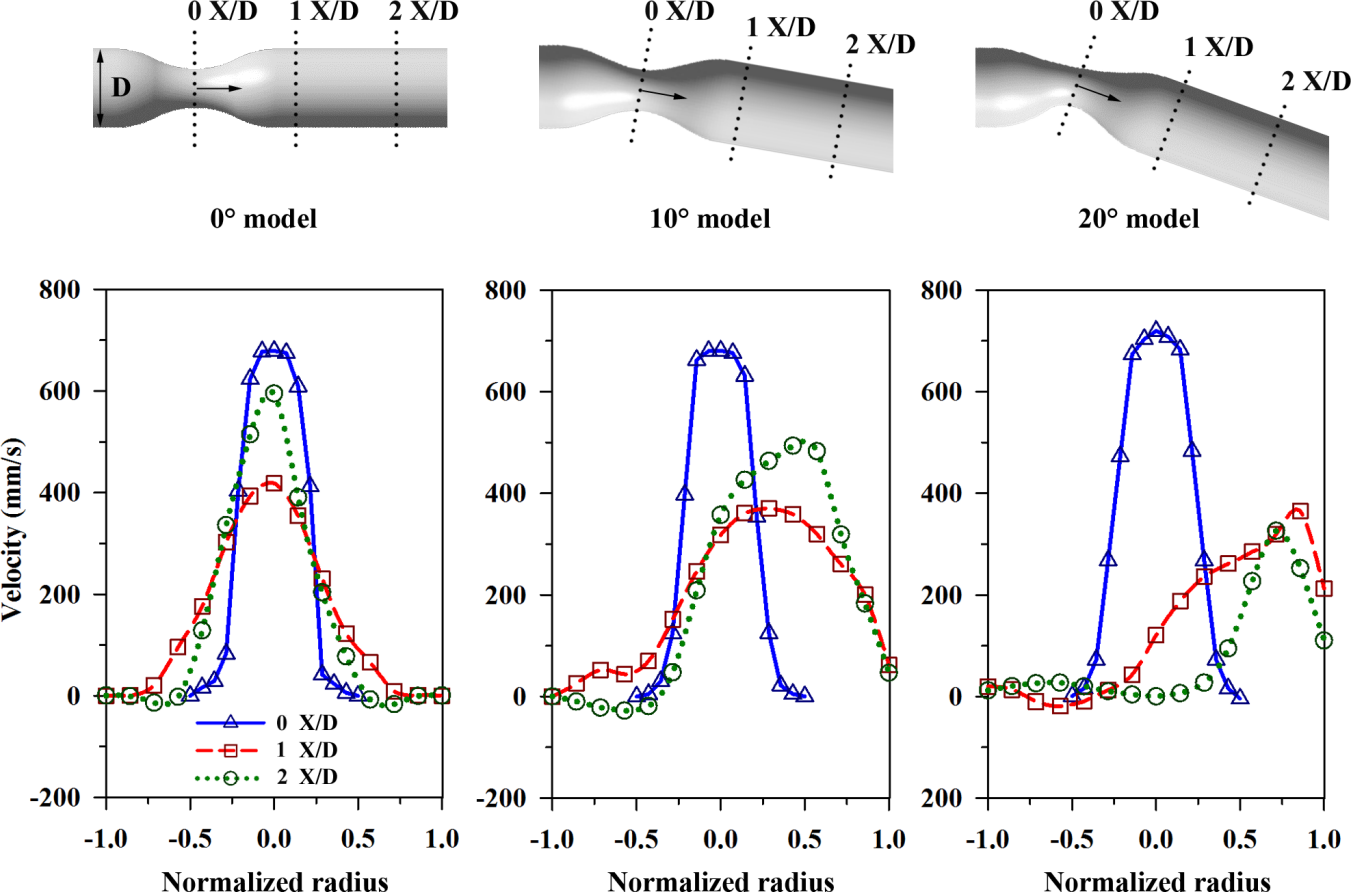
Veloicty distributions in the channels with and without curvature. The positions, where data were extracted, are marked on each model (upper panel) as 0 X/D, 1 X/D, and 2 X/D. The velocity distributions (lower panel) are presented for three types of channels at φ = 0.5 and Re = 160.

For further comparisons, velocity profiles were extracted from the vector fields. **Fig 7** shows the velocity profiles at different positions with respect to the flow stream of the channel (X/D = 0, 1, and 2) at Re = 160 and φ = 0.5. The bottom of **Fig 7** shows the extracted velocity profiles for the three models. The horizontal axis indicates the radial positions of the tube, and the vertical axis indicates the velocities in mm/s.

At 0 X/D, the velocity profiles have the highest values and are symmetrical along the longitudinal center axis for all vessel models. The pulsatile flow at φ = 0.5 has a recirculation zone around 2 X/D after passing the stenosis, which makes the velocity profile sharper at this point with higher values than those at 1 X/D for the case of the 0° and 10° channels. In the 10° model, the peak velocities at 1 X/D and 2 X/D occur at normalized radii of 0.25 and 0.5, respectively. In the 20° model, the peak velocities occur at normalized radius of 0.75 and 1, respectively. This result implies that the centrifugal force in bent stenosed tube induces that flows are skewed towards the outer curvature wall, and the degree of skewness of the velocity profile becomes more pronounced as the bent angle increases. The recirculation region near the outer curvature induces energy losses and a local pressure gradient. Furthermore, the fluid elements face the outer wall surface and are influenced by the local positive pressure gradient on this side. The flow velocity then decreases due to inertia. Therefore, the axial velocity profile is skewed toward the outer curvature wall and it loses its symmetrical shape.

In some previous studies, the pulsatile flow behaviors in the curved geometry were analyzed. Unlike the symmetric velocity profile in a straight pipe, a high axial velocity along the outside wall and the low velocity (sometimes flow reversal) near the inside wall in the curvature of arteries [35]. Jung, J., et al. (2006) and Timité, B., et al. (2010) reported that the secondary flow appears and the axial velocity isshifted towards the outside curvature wall under the effect of centrifugal force [33, 36]. A numerical study on curved pipe with sudden expansion shows the similar trend compared with the experiment results conducted in this study [37]. In **S3 Fig**, the centrifugal force is affected by the curvature of the channel by comparing simulation data in curved channel without or with stenosis. The absolute values of the case with stenosis are higher almost 10 times than that of the case without stenosis. Although the strength of WSS is different depending on the existence of stenotic geometry, the similar WSS trend explains the effect of curvature contributing to centrifugal force.

**Fig 8:**
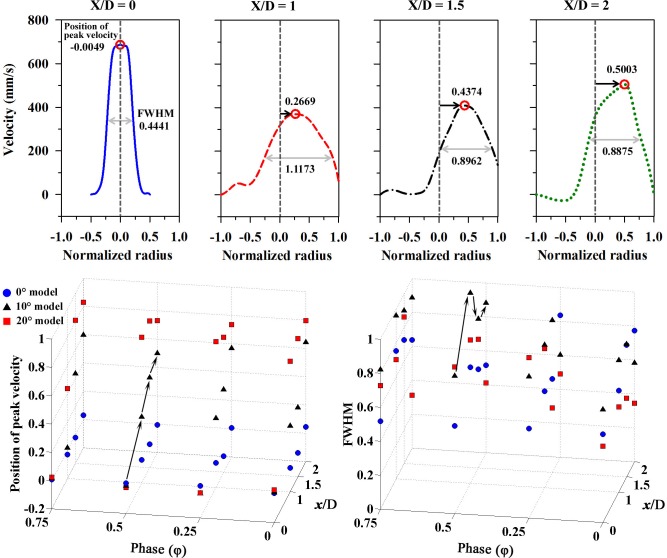
The positions of peak velocity and full width at half maximum (FWHM) for the 10° bent model (upper panel). The position of peak velocity and FWHM represent the level of skewness from the center axis and the bluntness of flow profile, repectively. These velues are represented for all models (lower panel) with respect to the phases (φ = 0, 0.25, 0.5, and 0.75) and positions (X/D = 0, 1, 1.5, and 2).

**Fig 8** represents the positions of the peak velocity and the values of full width at half maximum (FWHM) with respect to the phases and position of the flow stream for all models at Re = 160. The upper graphs depict these values on the velocity profile at four different positions in the 10° model. The position of peak velocity occurs closer to the outer curvature wall (normalized radius = 1.0) as X/D goes from 0 to 2. In the 0° model, the positions of the peak velocity are slightly varied at X/D = 2 due to circulation zone, but the differences are still slight compared to the 10° and 20° models. In these two models, the positions of peak velocity tend to be more skewed toward the outer curvature wall at the further downstream, regardless of the phases.

FWHM shows a trend in terms of the phases of the pulsatile flow (φ = 0, 0.25, 0.5, and 0.75). The variation of the width of the streamlines reflects the acceleration of the flow by the pulse, and the FWHM values are thus relatively high after the acceleration phase (φ = 0.5). In regard to the extracted positions (X/D = 0, 1, 1.5, and 2), the FWHM seems to be irregular in all models because the pulsatile flow circulation has affects the velocity distribution repeatedly along the longitudinal direction. However, in the 20° model, the FWHM is generally lower downstream (X/D = 2) due to the narrower main stream as result of the centrifugal force. A previous study confirms that there is a difference between the steady flow and pulsatile flow in regard to the secondary flow. This means that several modes of pulsatile flow are superposed on the steady flow component [17].

The stenosis in the curved tube results in redistribution of the secondary flow at downstream of the stenosis. The inviscid core region tends to move the location of the maximum axial velocity to the outer wall, while viscous effects are confined to a region close to the wall. This makes the maximum axial velocity shift towards the outer wall [15, 17]. The direction of the velocity vector is no longer parallel to the center line of the curved channel [25], which confirms that increasing the bend angle leads to a loss of symmetry of the axial velocity profiles and the maximum value is skewed toward the outer wall.

### Shear strain distribution in stenosed channels

**Fig 9:**
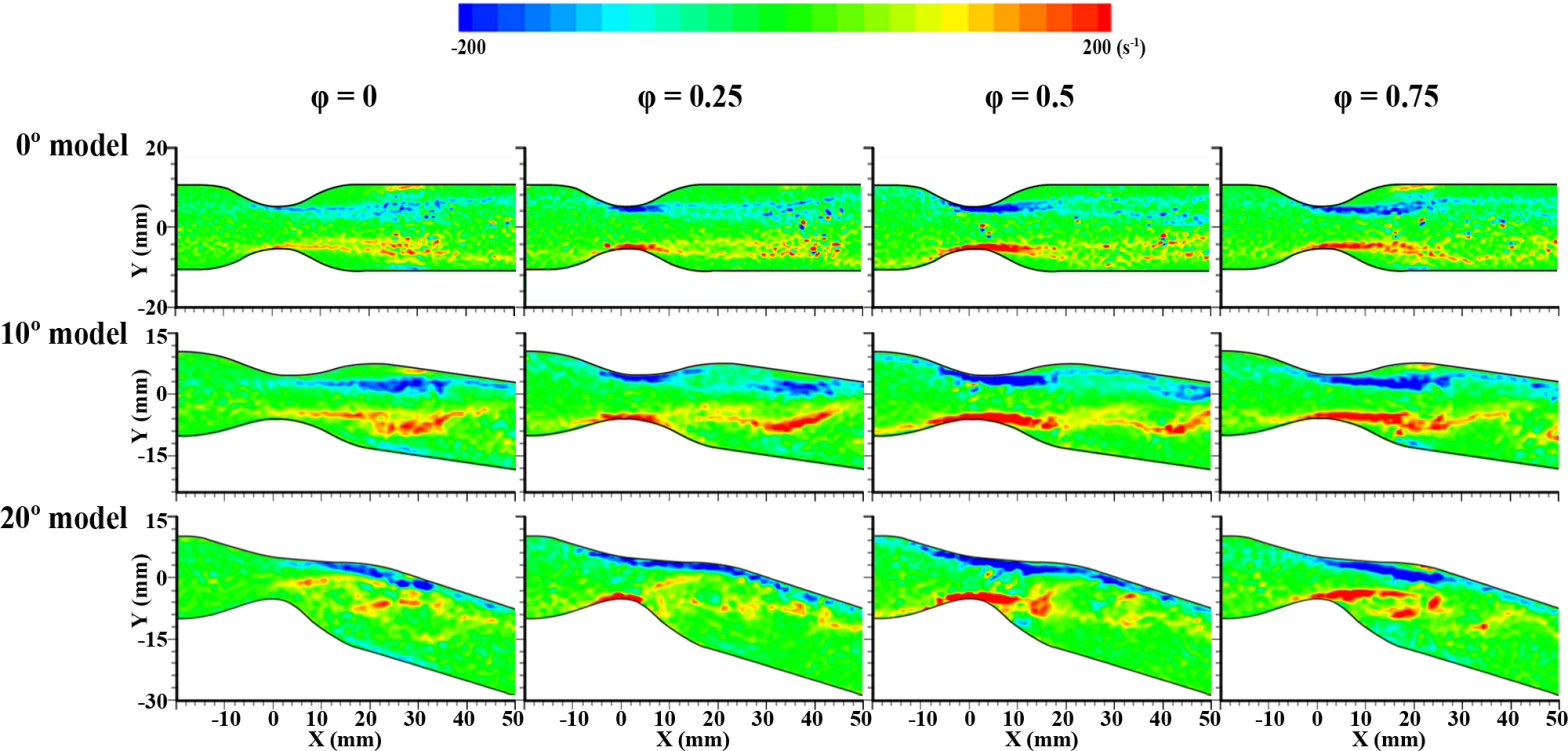
The comparisons of shear strain distributions in all stenosed models at four representative phases (φ = 0, 0.25, 0.5, and 0.75). From top to bottom panel, each indicates the case of 0° model, 10° bent model and 20° bent model.

**Fig 9** illustrates the distribution of the shear strain at four representative phases in each model. In the 0° model, a zone with high shear strain is mostly observed around the stenosed part, and it is intensified at φ = 0.5. A zone with locally weak shear strain at the wall is caused by vortices and moves along the flow stream during the deceleration phase from φ = 0.75 to 0. In the 10° model, a zone with high shear strain also appears around the stenosed channel, as well as near the outer curvature wall during specific deceleration phases (φ = 0.5 and 0.75).

In the 20° model, high shear strain occurs at the outer curvature over a wide longitudinal area (X = 0 to 30 mm), in contrast to the other two cases. Especially, this wide zone at the outer curvature is maintained in all phases. At φ = 0, relatively weak shear strain is observed on the opposite side at the inner curvature wall. When the recirculation flow at the inner curvature impinges the flow at the outer wall of the curved channel, highly accelerated flow occurs at the outer curvature. Consequently, WSS is higher at the outer side of the channel than elsewhere [4]. This behavior is different from that of a straight stenosed tube [16].

To estimate 3-dimensional flow characteristics from the present results, results obtained from numerical simulations were compared with the experimental results (**S1 Fig**). The velocity distributions and WSS distributions for experimental and numerical results are quite similar (**Figs 3 and 9**). This phenomenon is similarly observed in the different models. In the simple curved tube, the WSS at the outside wall is significantly higher than on the inside curvature [35, 36]. The simulation results for pulsatile flows in curved tube with coarctation represented that the maximal WSS is located at the obstructions and the presence of recirculation zone results in locally reduced WSS at downstream of the obstructions. In addition, the pressure is largely reduced as flow passes through the obstruction [15]. Hye, M. A. and M. C. Paul (2015) also conducted computational study for curved tube with stenosis under the steady flow. In their study, the maximum pressure drop and WSS is bigger in the 120° curved model than in the straight model. Additionally, the increase in WSS can be observed just prior to the throat of the stenosis [16].

High WSS from ascending to center part of stenosis exists at φ = 0.25–0.75 regardless of the curvature of the channel as shown in **Fig 9**. Especially, the 20° model has significantly high WSS along the outer curvature wall. **S4 Fig** illustrates the distribution of WSS in the 20° model depending on the Reynolds number. As Reynolds number increases, overall flow patterns are similar but the intensity of WSS grows. Dolan, J. M., et al. (2013) reported that high WSS induces a unique endothelial state compared to baseline WSS [38] and this may be important for the adaptive and pathological remodeling [4]. Specifically, WSS induces biologic effects in endothelial cells (ECs) that can affect the crucial balance between cap-reinforcing matrix synthesis and breakdown [39]. The region of high WSS stimulates the ECs to produce nitric oxide that might suppress synthetic smooth-muscle proliferation (matrix synthesis) and stimulate secreting metalloproteinases (matrix degradation) [40]. This effect might enhance the thinning of fibrous cap with the effect of cyclic strain which is generated by pulsed pressure and is related to vulnerability to atherosclerosis [41, 42]. Then, a plaque ruptures when the wall stress in the lesion exceeds the fracture stress of the fibrous cap [39]. Results from Gijsen, F., et al. (1999) supported the mechanism that upstream plaque regions exposed to high WSS are more prone to rupture [32, 41–43]. Finally, plaque rupture also increases the risk of heart attacks and stroke [10, 44, 45]. In this study, the distribution of WSS is highly affected by the degree of curvature and the intensity is affected by the frequency of pulsatile flow (Reynolds number). Therefore, the geometry also can be considered as an important factor of progression of stenosed vessel.

### 3D velocity distribution

**Fig 10:**
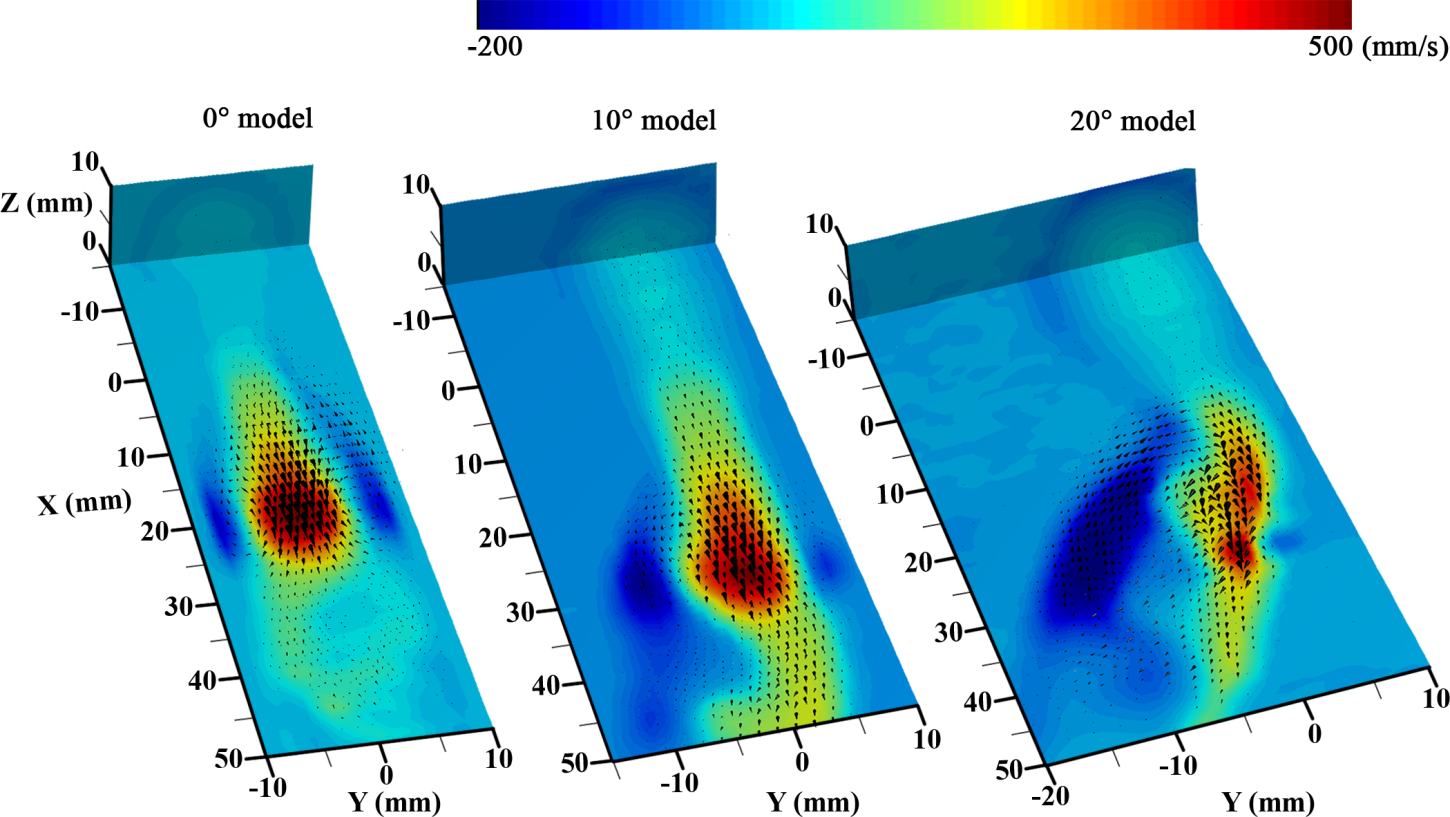
The 3D flow patterns converted from a stack of 2D velocity data in the 0° model (left-side), 10° bent model (middle), and 20° bent model (right-side). The velocity contoured plane is the center measured X-Y plane (Z = 0 mm), and the black arrows represent the 3-dimensional velocity vector distributions. To represent the velocity values in Z-coordinate, results from the numerical simulation were added in a stack of 2D vectors obtained from the experiments.

A stack of 2D velocity fields was obtained to investigate the 3D flow structure. **Fig 10** shows the 3D velocity distributions at φ = 0 for the different models. Since 2D velocity fields were experimentally obtained by changing the laser sheet along the Z direction, there is no third velocity (w) information. To compensate w-velocity component in the 3D flow structure, the w-velocity information in Z-coordinate in numerical simulation results were combined with experimental planar vectors (**S5 Fig**). In the diastole phase, a vortex ring is created by hydrodynamic deceleration and occurs after the stenotic region [46].

Since the 0° model is a symmetrical and straight channel, the vortex structure is also symmetrical with a doughnut-shaped ring. In the case of the 10° model, the axial velocity of the flows moves towards the outer curvature wall and the vortex structure becomes distorted, but a ring-shaped vortex with a thick side still occurs at the inner curvature wall. The forward flow in the 20° channel is the most skewed towards the outer curvature wall for all Z values. Accordingly, the backward flow is strong, and the recirculation region occupies a rather wide area at the inner curvature.

**Fig 11:**
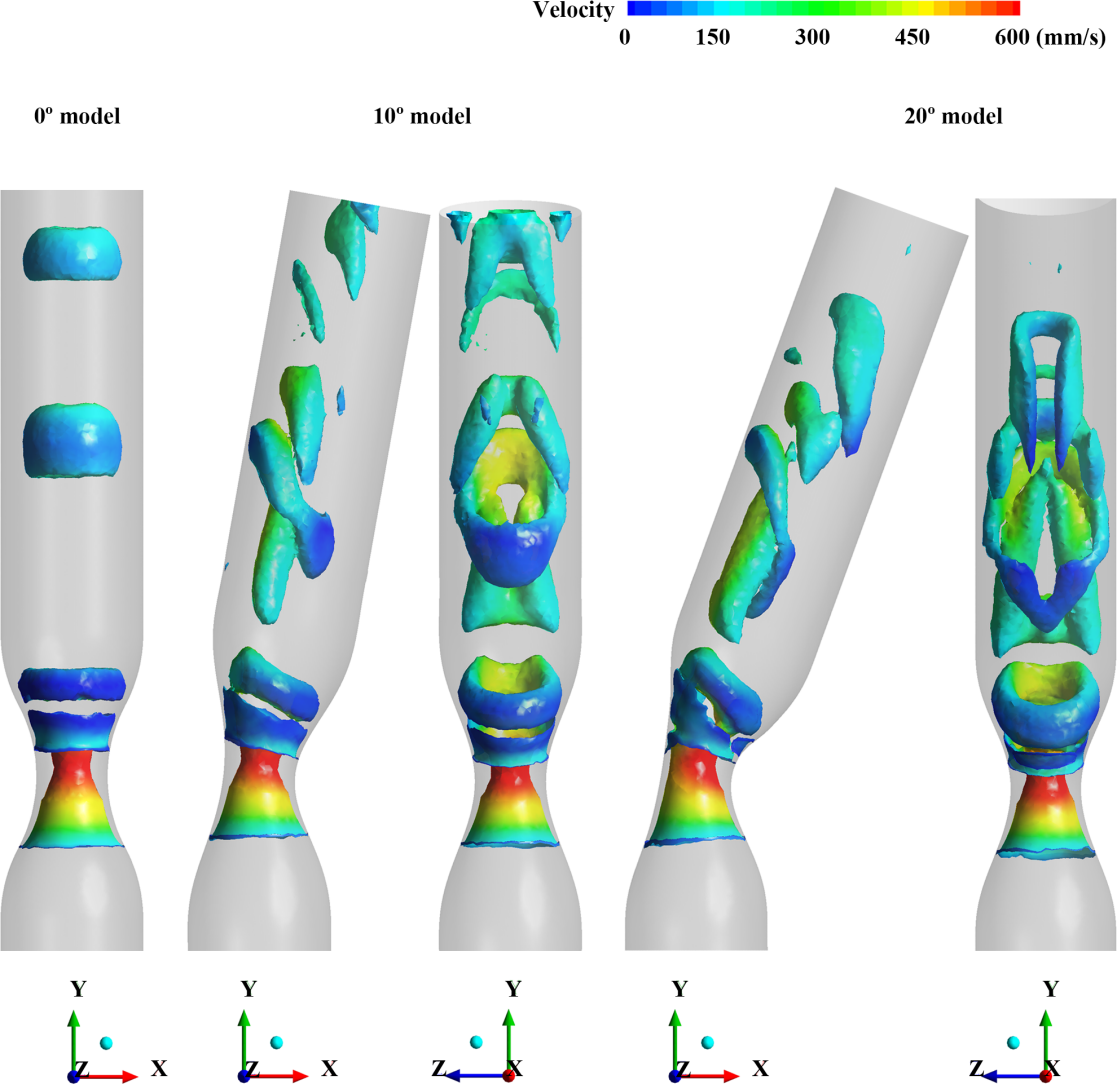
The 3-dimensional flow structures in the cases of 0°, 10° and 20° models by using isosurfaces of Q-criterion (150 s^-2^) from the numerical simulation.

To validate the 3D flow structure, the isosurface of Q-criterion obtained by numerical simulation was used for displaying vortex in the stenosed models (**Fig 11**) [47]. It shows similar trend with the experimental results. In the case of 0° model, the vortex looks like a symmetrical vortex-ring. As the degree of curvature increases from 10° to 20°, the vortex ring is more skewed to the outer curvature wall. Additionally, it is tilted at an angle from the cross section of the channel and stretched longitudinally due to the higher velocity at the outer wall resulting from the centrifugal force.

## Conclusion

Pulsatile flow characteristics in 3D curved stenosed channels were analyzed using time-resolved PIV. Asymmetrical flow structures occurred in the curved models, whereas symmetrical flow structures occurred in the straight model. The centrifugal force resulting from the curved geometry induces skewed flow toward the outer curvature wall. As the bend angle increased, the axial velocity became more skewed more toward the outer wall. The local pressure gradient caused by the skewed velocity has an influence on the reverse flow at the inner curvature wall, as well as the velocity magnitude and direction of the main flow stream.

The skewing became more pronounced as the Reynolds number decreased and the streamline progressed further downstream. As a result, the pulsatile flow is considerably affected by the curved geometry factor rather than steady flow condition. The 3D structures of the vortex are also very different depending on curvatures. In all cases, high WSS appears around the stenosis apex. However, in the 20° channel, noticeably high WSS also occurred at the outer wall. The normal wall shear stress in the healthy human aorta maintains from 1 to 2 Pa (shear strain; 80 to 160 s^-1^) [39, 48]. From the shear strain distribution in **Fig 9**, the value of shear stress marked as blue and red color is obtained around 2.5 Pa (shear strain; around ± 200 s^-1^). One of the most interesting observations is that high WSS occurred not only near the stenosed throat but also at the outer wall. These experimental results were matched with simulation results. Asymmetrical WSS in a curved stenosed channel may be associated with a thinning fibrous cap, instability of the plaque, and eventual rupture associated with increased risk of heart attacks or stroke.

## Supporting information

S1 Fig Numerical data representing the distribution of velocity (upper panel) and wall shear stress (lower panel). Waveforms of experimental data and fitted mass flowrate (the upper right side).
(TIF)

S2 Fig Comparison between of experiment and simulation data. Velocity profiles at upstream of stenosis obtained by experiment and simulation results (left side). The difference between the input flow rate and measured flow rate (Q_experiment_—Q_simulation_) is depicted in the Bland–Altman plot with respect to their average value (right side). Bold line and dashed lines denote the mean value and 95% limits of agreement, respectively.
(TIF)

S3 Fig The distribution of WSS at the wall of channel in the case of 20° model (a) without stenosis (b) with stenosis.
(TIF)

S4 Fig The comparisons of shear strain distributions in Reynolds number conditions at the bottom and peak phase velocity (φ = 0 and 0.5). From top to bottom panel, each indicates the condition of Re = 160, 260 and 360.
(TIF)

S5 Fig The third component velocity distributions (*w*) on the X-Y plane were subtracted using the numerical simulation data from center to the top in Z-direction. (TIF)

S1 Movie The moving images of contoured velocity vector field at Re = 160 in the 0° model. (AVI)

S2 Movie The moving images of contoured velocity vector field at Re = 160 in the 10° model. (AVI)

S3 Movie The moving images of contoured velocity vector field at Re = 160 in the 20° model. (AVI)
