# Supplementary figures and images for: Characteristics of pulsatile flows in curved stenosed channels
Source: PLoS One. 2017 Oct 19;12(10):e0186300. doi: 10.1371/journal.pone.0186300 (PMC5648160; doi:10.1371/journal.pone.0186300)

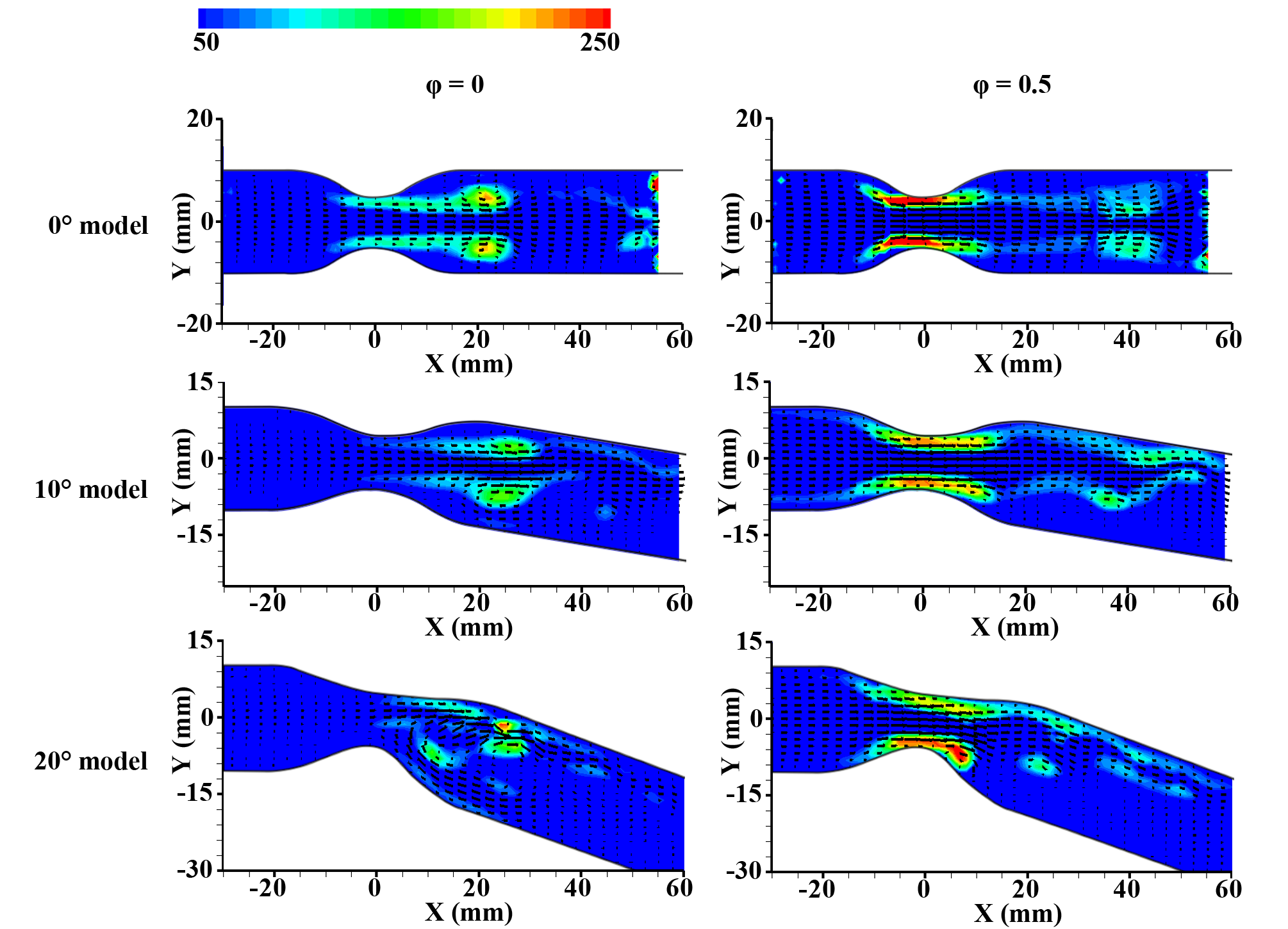

Supplement: S1 Fig — Waveforms of experimental data and fitted mass flowrate (the upper right side). (TIF) [file pone.0186300.s001.tif]

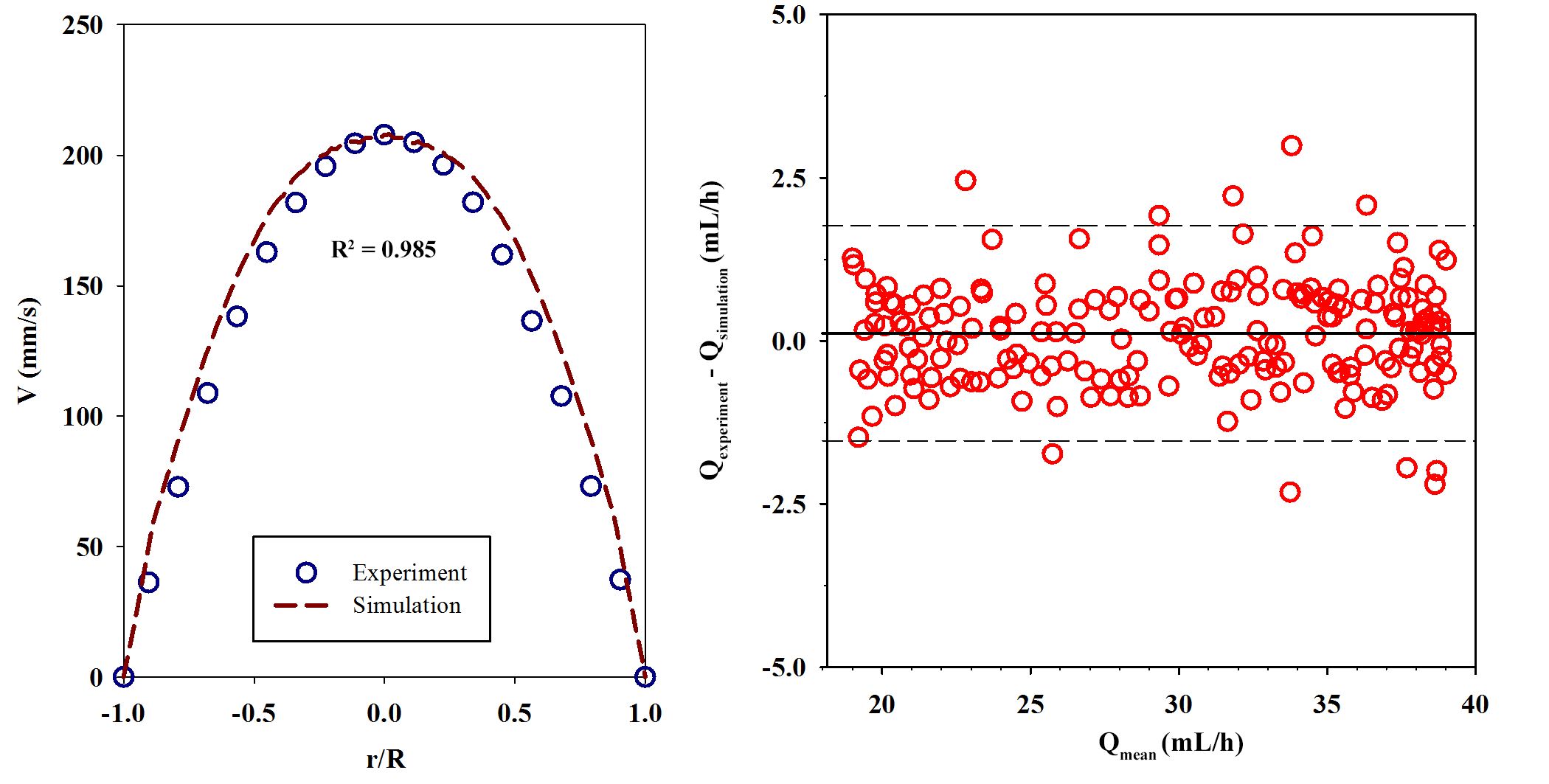

Supplement: S2 Fig — Velocity profiles at upstream of stenosis obtained by experiment and simulation results (left side). The difference between the input flow rate and measured flow rate (Qexperiment—Qsimulation) is depicted in the Bland–Altman plot with respect to their average value (right side). Bold line and dashed lines denote the mean value and 95% limits of agreement, respectively. (TIF) [file pone.0186300.s002.tif]

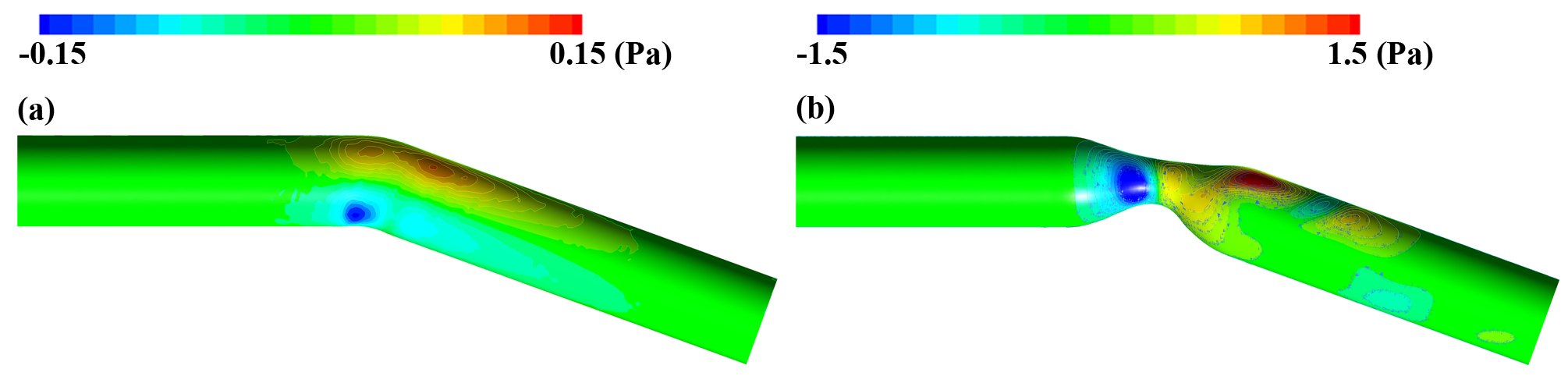

Supplement: S3 Fig — The distribution of WSS at the wall of channel in the case of 20° model (a) without stenosis (b) with stenosis. (TIF) [file pone.0186300.s003.tif]

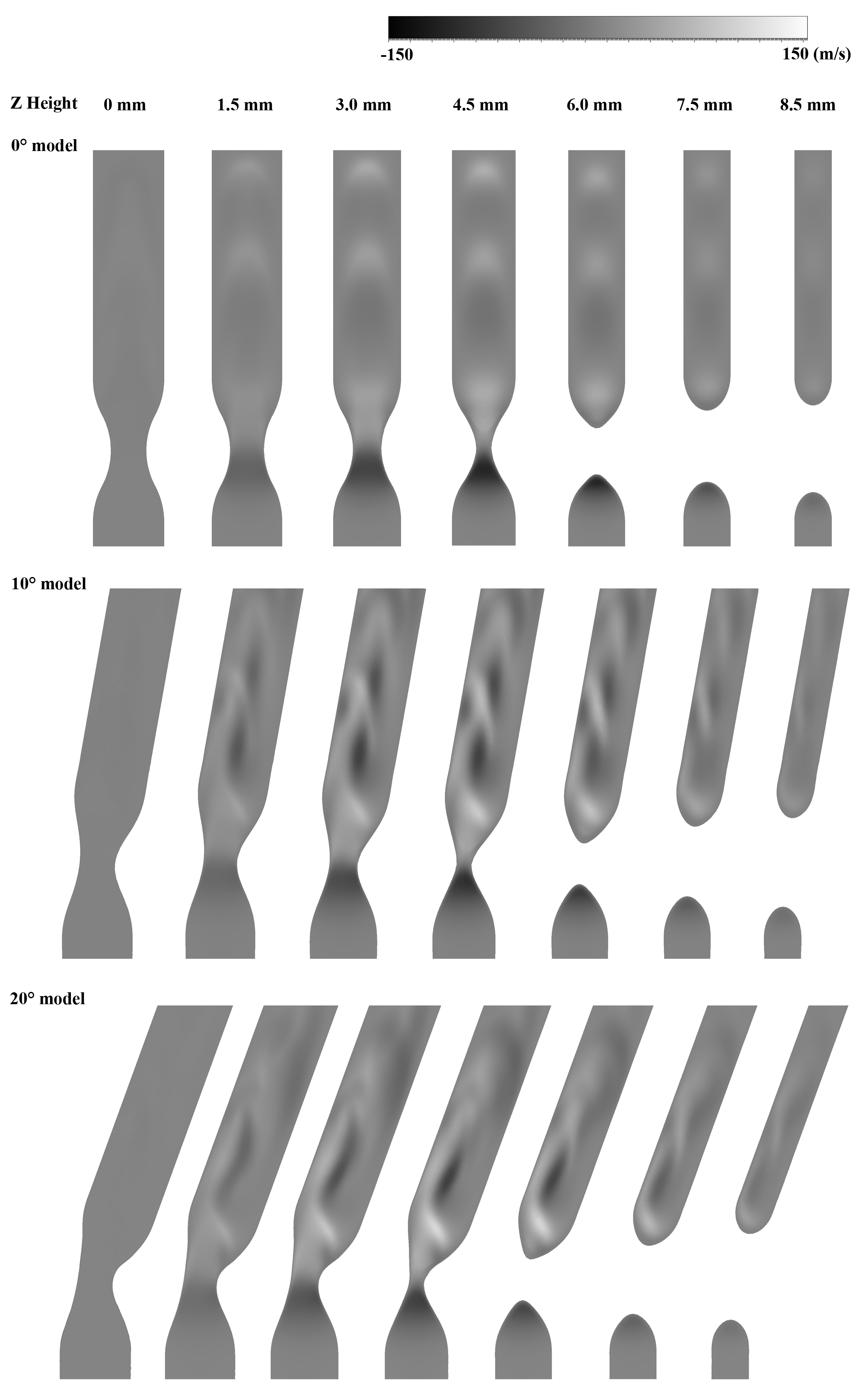

Supplement: S4 Fig — From top to bottom panel, each indicates the condition of Re = 160, 260 and 360. (TIF) [file pone.0186300.s004.tif]
